# Functional Characterization of Two Glutamate Dehydrogenase Genes in *Bacillus altitudinis* AS19 and Optimization of Soluble Recombinant Expression

**DOI:** 10.3390/cimb47080603

**Published:** 2025-08-01

**Authors:** Fangfang Wang, Xiaoying Lv, Zhongyao Guo, Xianyi Wang, Yaohang Long, Hongmei Liu

**Affiliations:** 1 Biochemistry Teaching and Research Section, School of Basic Medical Sciences, Guizhou Medical University, Anshun 561113, China; wangfangfang24680@163.com; 2 Engineering Research Center of Medical Biotechnology, School of Biology and Engineering, Guizhou Medical University, Anshun 561113, China; 13985018141@163.com (X.L.); asd237215963@126.com (Z.G.); wxyumost@163.com (X.W.); 3 Key Laboratory of Biology and Medical Engineering, Immune Cells and Antibody Engineering Research Center of Guizhou Province, Guizhou Medical University, Anshun 561113, China; 4 Engineering Research Center of Health Medicine Biotechnology of Institution of Higher Education of Guizhou Province, Guizhou Medical University, Anshun 561113, China; 5 Laboratory Animal Center of Guizhou Medical University, Anshun 561113, China

**Keywords:** glutamate dehydrogenase, *Bacillus*, soluble expression, bioinformatics

## Abstract

Glutamate dehydrogenase (GDH) is ubiquitous in organisms and crucial for amino acid metabolism, energy production, and redox balance. The *gdhA* and *gudB* genes encoding GDH were identified in *Bacillus altitudinis* AS19 and shown to be regulated by iron. However, their functions remain unclear. In this study, *gdhA* and *gudB* were analyzed using bioinformatics tools, such as MEGA, Expasy, and SWISS-MODEL, expressed with a prokaryotic expression system, and the induction conditions were optimized to increase the yield of soluble proteins. Phylogenetic analysis revealed that GDH is evolutionarily conserved within the genus *Bacillus*. GdhA and GudB were identified as hydrophobic proteins, not secreted or membrane proteins. Their structures were primarily composed of irregular coils and α-helices. SWISS-MODEL predicts GdhA to be an NADP-specific GDH, whereas GudB is an NAD-specific GDH. SDS-PAGE analysis showed that GdhA was expressed as a soluble protein after induction with 0.2 mmol/L IPTG at 24 °C for 16 h. GudB was expressed as a soluble protein after induction with 0.1 mmol/L IPTG at 16 °C for 12 h. The proteins were confirmed by Western blot and mass spectrometry. The enzyme activity of recombinant GdhA was 62.7 U/mg with NADPH as the coenzyme. This study provides a foundation for uncovering the functions of two GDHs of *B. altitudinis* AS19.

## 1. Introduction

Glutamate dehydrogenase (GDH) is a ubiquitous metabolic enzyme that plays a crucial role in regulating energy metabolism, intracellular redox homeostasis, amino acid metabolism, and signal transduction in the tricarboxylic acid cycle (TCA) [1]. The two main pathways for glutamate synthesis are the GDH-catalyzed reaction and the reaction co-catalyzed by glutamine synthetase (GS) and glutamate synthase (GOGAT). In both pathways, the carbon backbone of glutamate, α-ketoglutarate, originates within the TCA. Thus, glutamate biosynthesis connects the carbon and nitrogen cycles [2]. GDH catalyzes the interconversion of glutamate to α-ketoglutarate and ammonia. This links amino acid metabolism to the TCA [3].

Different strains exhibit variations in the composition, number, and function of GDH-encoding genes. In the Gram-negative bacterium *Escherichia coli*, GDH is encoded solely by the *gdhA* gene [4,5], which is crucial for ammonia assimilation and glutamate synthesis [6]. In contrast, the Gram-positive model strain *Bacillus subtilis* 168 encodes two GDH proteins, RocG and GudB, through the *rocG* and *gudB* genes [7]. These two proteins share 74.8% amino acid similarity [8]. During normal growth, RocG functions as the dominant glutamate-degrading enzyme [9] and additionally regulates both GltC-mediated glutamate synthesis and antibiotic resistance [10]. The *gudB* gene expression is strictly dependent on *rocG* inactivation, exhibiting activation only when *rocG* function is impaired [2,7]. In *Corynebacterium glutamicum* F343, two GDH-encoding genes (*gdhA* and *gdhB*) are present, with GdhA involved in ammonia assimilation and GdhB in glutamate catabolism [11]. Garcia LN et al. reported that *Bacillus altitudinis*, *Bacillus safensis*, and *Bacillus tequilensis* possess only the glutamate dehydrogenase GudB [12]. In *Clostridium difficile*, GDH serves as an important clinical diagnostic marker, because it is highly conserved in both toxigenic and non-toxigenic strains, and a negative GDH result in stool can rule out *C. difficile* colonization or infection with 100% certainty [13]. In *Streptococcus suis* type 2, GdhA is essential for cell growth and pathogenicity; Chittick et al. demonstrated experimentally that GDH supplies nitrogen and energy precursors to the strain, thereby maintaining intracellular redox balance and rapid proliferation, and is directly involved in pathogenesis [14]. In *Streptococcus thermophilus*, GdhA is associated with aromatic compound synthesis [15]. In *Salmonella*, GdhA provides resistance to oxidative damage; it catalyzes the assimilation of ammonium into glutamate, thereby boosting intracellular glutathione synthesis and conferring resistance to oxidative stress [16]. In *Streptococcus pneumoniae*, GdhA is necessary for virulence under high-temperature acclimatization. Experimental results showed that maintaining the glutamate-α-ketoglutarate flux ensures energy supply and cell-wall stability under high-temperature conditions [17].

The *Bacillus altitudinis* AS19 strain was isolated from the rhizosphere of *Paris polyphylla* var. *yunnanensis* in our laboratory. This strain exhibits the characteristics of secreting hydroxamate-type siderophores, ammonia production, and inorganic phosphate solubilization. The fermentation supernatant of this strain strongly inhibited *Candida albicans* under iron-deficient conditions [18]. However, the inhibitory activity decreased when iron ions were added, suggesting that the siderophore may have antimicrobial activity. To characterize the nutritional regulation of siderophore biosynthesis, we assessed strain AS19’s response to different amino acid supplements. It was found that when 1.4 g/L glutamic acid was added to the SA iron-deficient medium, strain AS19 could not grow, while other strains grew normally. This phenomenon did not occur in the LB medium. Therefore, it was hypothesized that strain AS19 had abnormal glutamate metabolism in the SA iron-deficient medium.

Genome-wide analysis revealed the presence of two GDH-encoding genes (*gdhA* and *gudB*) in strain AS19. RT-qPCR results showed that iron ions affected the expression of these two genes. While glutamate dehydrogenase function is well established, the *gdhA-gudB* pairing remains rarely reported in *Bacillus* strains, suggesting potential functional specialization requiring characterization. In this study, the two GDHs of *B. altitudinis* AS19 were analyzed using bioinformatics tools, and conditions for soluble recombinant expression were optimized. The evolutionary relationship of GDH within the genus *Bacillus* was investigated, and its physicochemical properties and spatial structure were predicted. Enzyme activity was also measured. This work lays the foundation for elucidating the biological functions of these GDHs.

## 2. Materials and Methods

### 2.1. Strains and Plasmids

*Bacillus altitudinis* AS19 was isolated from the rhizosphere soil of *Paris polyphylla* var. *yunnanensis* by our research group. The pET28a expression vector was stored in our laboratory; *Escherichia coli* BL21 (DE3) was used as the host strain.

### 2.2. Enzymes and Reagents

All commercial reagents and kits were obtained from the following sources: restriction endonucleases from SibEnzyme (Novosibirsk, Russia); DNA polymerase, DNA markers, and protein molecular weight markers from Beijing Kangrun Chengye Biotechnology Co., Ltd. (Beijing, China); bacterial DNA extraction kits (including genome extraction, plasmid mini-preparation, and DNA purification/recovery kits) from Tiangen Biotech (Beijing, China); kanamycin, Rainbow 180 protein molecular weight marker, IPTG, and SDS-PAGE reagents from Beijing Solarbio Technology Co., Ltd. (Beijing, China); Western blot antibodies from Beijing Quanshijin Biotechnology Co., Ltd. (Beijing, China); PVDF membranes from Merck Millipore (Burlington, MA, USA); and Ni-NTA resin from MedChemExpress (Monmouth Junction, NJ, USA). All other chemicals were analytical grade and commercially obtained.

### 2.3. Medium

LB liquid and solid media were prepared by dissolving 5 g of tryptone, 2.5 g of yeast extract, and 5 g of sodium chloride in 500 mL of deionized water. For solid medium, 7.5 g of agar was added. The media were sterilized by autoclaving at 121 °C for 25 min.

### 2.4. Bioinformatics Analysis of Glutamate Dehydrogenase Genes and Protein Characterization

The complete genome sequences of 14 *Bacillus* type strains, representing major phylogenetic groups of the genus, were downloaded from the NCBI database. Homologs of the *gdhA* and *gudB* genes were identified using BioEdit, and their corresponding amino acid sequences were extracted. These sequences were aligned using the ClustalW algorithm in MEGA 11.0. A phylogenetic tree of glutamate dehydrogenases was constructed using the Neighbor-Joining method with 1000 bootstrap replicates. The glutamate dehydrogenase from *Theobroma cacao* was used as an outgroup. Similarly, *16S rRNA* sequences from the same strains were aligned and used to construct a separate phylogenetic tree, with the *16S rRNA* of *Escherichia coli* as the outgroup. These analyses provide insights into the evolutionary relationships within the *Bacillus* genus.

Protein characterization was performed using multiple bioinformatics tools: (1) ProtParam on ExPASy server [19] (https://www.expasy.org/resources/protparam) for determining molecular weight, theoretical pI, and other physicochemical properties; (2) DeepTMHMM-1.0 for transmembrane domain prediction; (3) SignalP 6.0 [20] with default parameters for signal peptide analysis; (4) SOPMA accessed via NPS@ [21] (https://npsa-pbil.ibcp.fr/cgi-bin/npsa_automat.pl?page=/NPSA/npsa_sopma_f.html) for secondary structure prediction using a consensus of four independent methods; (5) SMART (https://smart.embl.de/) [22] for comprehensive domain architecture analysis; and (6) SWISS-MODEL (https://www.expasy.org/search/swiss-model) [23] for homology-based tertiary structure prediction, with model quality assessed by QMEAN and GMQE scores.

### 2.5. Primer Design and Synthesis

Specific primers for amplifying *gdhA* and *gudB* genes were designed using SnapGene 7.0, incorporating appropriate restriction enzyme sites and protective bases (Table 1). All oligonucleotides were synthesized by Shanghai Biotechnology Co., Ltd. (Shanghai, China).

### 2.6. Target Gene Amplification

The *gdhA* and *gudB* genes were amplified from *B. altitudinis* AS19 genomic DNA using high-fidelity PCR. The reaction mixture (50 µL) contained 25 µL of 2 × SuperNova PCR Mix, 2.5 µL each of forward and reverse primers, 1 µL of template DNA, and nuclease-free water to adjust the volume. The thermal cycling conditions were as follows: initial denaturation at 98 °C for 3 min; 32 cycles of denaturation at 98 °C for 10 s, annealing at 58 °C for 15 s, and extension at 72 °C for 45 s; and a final extension at 72 °C for 7 min. The PCR products were analyzed by 1% agarose gel electrophoresis.

### 2.7. Recombinant Plasmid Construction and Verification

PCR products encoding the full-length glutamate dehydrogenase genes *gdhA* and *gudB* were purified using a DNA purification and recovery kit provided by Tiangen Biotech (Beijing) Co., Ltd. (Beijing, China). The purified products were double-digested with the following restriction enzymes: *gdhA* with *Bam*H I and *Hin*d III, and *gudB* with *Bam*H I and *Eco*R I. The digested products were then ligated into the pET28a vector to construct the pET28a-*gdhA* and pET28a-*gudB* plasmids, respectively. The recombinant plasmids were transformed into *E. coli* BL21 (DE3) and plated on LB agar containing 100 µg/mL kanamycin. Single colonies were selected for PCR verification and plasmid extraction, followed by double digestion validation. Positive clones were sent to Shanghai Biotechnology Co., Ltd. (Shanghai, China) for sequencing. The expression construct was generated by inserting the target gene into pET-28a multiple cloning sites, producing recombinant protein bearing an N-terminal hexahistidine tag (0.84 kDa).

### 2.8. Soluble Expression Optimization and Purification of Recombinant GdhA and GudB Proteins

Single colonies of *E. coli* transformed with pET28a-*gdhA* and pET28a-*gudB* were inoculated into 50 mL of LB liquid medium containing 100 µg/mL kanamycin and cultured overnight with shaking. The seed cultures were then inoculated at a 1:100 ratio into 50 mL of LB liquid medium containing 100 µg/mL kanamycin. The cultures were grown at 37 °C with shaking at 200 r/min until the OD_600_ reached 0.6. IPTG was added to achieve final concentrations of 0.2, 0.4, 0.6, 0.8, and 1.0 mmol/L. The cultures were then incubated in a shaking incubator at 28 °C and 200 r/min for 12 h. SDS-PAGE was used to detect expression and determine the optimal IPTG concentration. With the optimal IPTG concentration, induction was performed at 16, 22, and 28 °C for 8, 12, 16, and 20 h, respectively, with a shaking speed of 200 r/min.

Cells were harvested by centrifugation at 12,000 r/min for 15 min at 4 °C after induction under optimized conditions. The cell pellets were resuspended in phosphate-buffered saline (PBS) and disrupted by sonication. The lysates were centrifuged again to separate the supernatant and pellet. The presence of the target protein in the supernatant was assessed to determine solubility. If the protein was expressed as inclusion bodies, strategies to enhance solubility included reducing IPTG concentration, lowering induction temperature, or shortening induction time.

Recombinant proteins were purified using Ni-NTA resin from MedChemExpress (MCE) (Shanghai, China) following the manufacturer’s protocol. Gradient imidazole elution was used to improve protein purity. The flow-through, wash, and eluate fractions were subjected to SDS-PAGE analysis.

### 2.9. Western Blot Analysis and Mass Spectrometry Identification

The expression products were separated by SDS-PAGE and transferred to a PVDF membrane. The membrane was blocked with TBST containing 5% skim milk powder. After washing three times with TBST, the membrane was incubated overnight at 4 °C with a mouse anti-His monoclonal antibody (1:5000 dilution). The membrane was then washed three times with TBST and incubated with HRP-conjugated goat anti-mouse IgG (1:10,000 dilution) for 1 h at room temperature with shaking. After three more washes with TBST, the membrane was developed with ECL reagent and imaged. The SDS-PAGE gel strips were then sent to a company for mass spectrometry confirmation.

### 2.10. Detection of Enzyme Activity of Recombinant Protein

Prior to the assay, all enzyme preparations were extensively dialyzed against PBS buffer (10 mM sodium phosphate, 150 mM NaCl, pH 7.4) using 10 kDa MWCO membranes (4 °C, 24 h with 3 buffer changes) to remove residual imidazole. Glutamate dehydrogenase activity was determined according to Zhao et al. [24] using two reaction systems in 3 mL final volume at 25 °C.

System A (Reductive Amination): 300 μL 1 M Tris-Cl (pH 8.0), 6 μL 0.5 M EDTA, 100 μL 1 M NH_4_Cl, 10 mM α-ketoglutarate (2.2 mg), 0.2 mM NADH/NADPH (0.25 mg), and 2 μL enzyme sample.

System B (Oxidative Deamination): 300 μL 1 M Tris-Cl (pH 8.0), 6 μL 0.5 M EDTA, 56.6 mM L-glutamate (25 mg), 0.2 mM NAD^+^/NADP^+^ (0.5 mg), and 2 μL enzyme sample.

Reactions were initiated by enzyme addition and monitored at 340 nm for 1 min. One unit (U) was defined as 1 nmol NAD(P)^+^ reduced or NAD(P)H oxidized min^−1^ mg^−1^ protein, calculated from initial linear-phase kinetics. Each system was analyzed in triplicate across three independent experiments, with protein concentrations determined by BCA assay using a Youke L4 spectrophotometer manufactured by Youke Biochemical Technology Co., Ltd. (Shanghai, China).

## 3. Results

### 3.1. Bioinformatics Analysis of GdhA and GudB

#### 3.1.1. Phylogenetic Analysis of Glutamate Dehydrogenase GdhA and GudB

**Figure 1:**
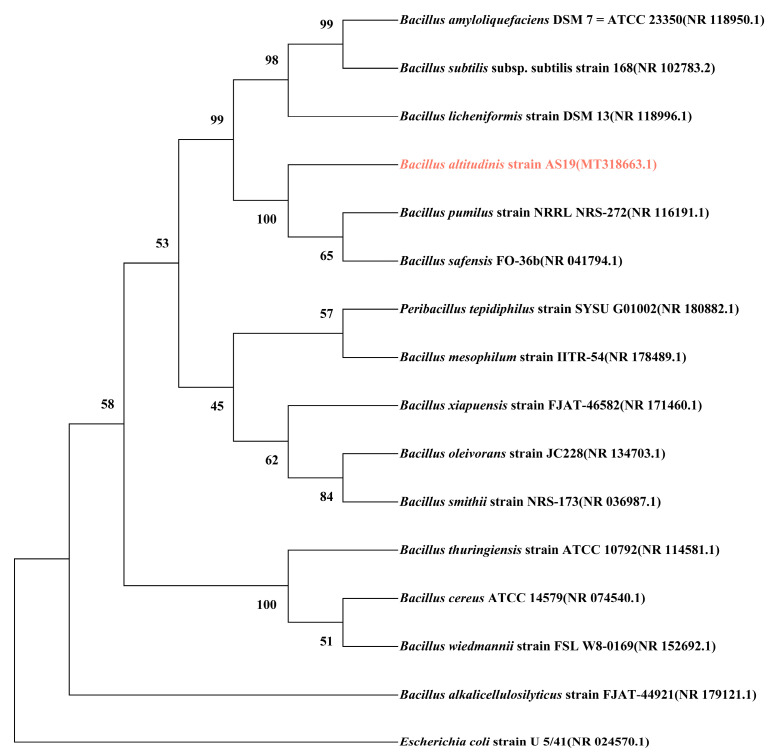
The phylogenetic tree constructed based on the *16S rRNA* sequences of 15 *Bacillus* species. The pink color represents *B. altitudinis* AS19. The numbers on each branch node indicate the bootstrap support percentage of the corresponding branch.

The phylogenetic tree based on *16S rRNA* gene sequences (Figure 1) revealed that *B. altitudinis* AS19 clustered closely with *B. pumilus* and *B. safensis*, forming a small clade with relatively close evolutionary relationships. Within this clade, *B. pumilus* and *B. safensis* exhibited a closer genetic proximity to each other than to AS19, suggesting that AS19 may possess distinct evolutionary traits despite its relatedness. Furthermore, this minor clade subsequently grouped with *B. amyloliquefaciens*, *B. subtilis*, and *B. licheniformis* into a larger cluster, indicating that AS19 shares a common evolutionary lineage with these well-characterized *Bacillus* species.

Glutamate dehydrogenases and their corresponding genes in 15 *Bacillus* strains were identified using BioEdit (Table 2). The results revealed notable differences in the composition and gene sequences of glutamate dehydrogenases among the strains. The GudB protein of *Bacillus pumilus* shares 99.76% sequence identity with that of *B. altitudinis* AS19, while *B. safensis* GudB is 100% identical to AS19 GudB. In general, strains with closer phylogenetic relationships tend to exhibit higher protein sequence similarity in their glutamate dehydrogenases.

**Figure 2:**
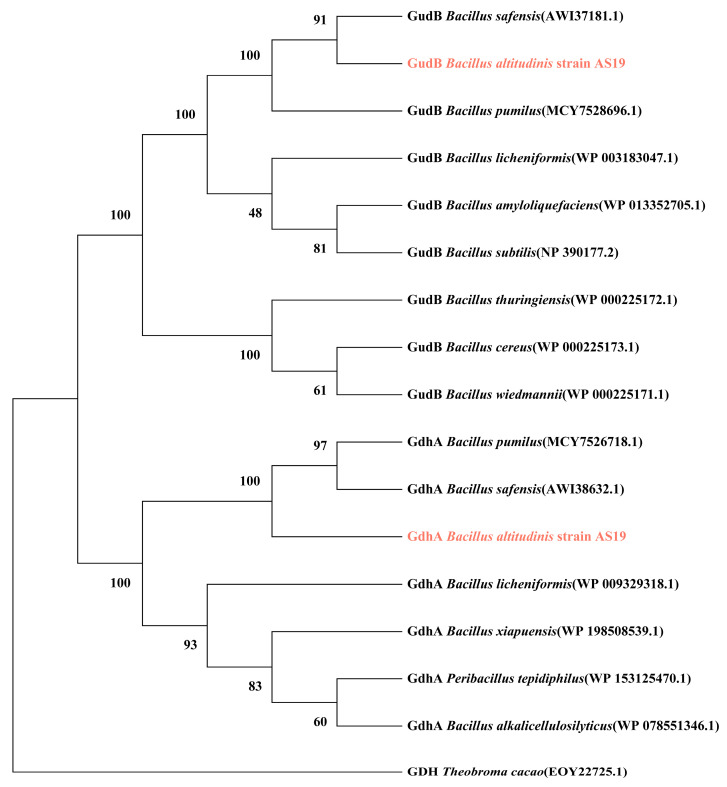
The phylogenetic tree of glutamate dehydrogenases GdhA and GudB. Pink represents the glutamate dehydrogenases of *B. altitudinis* AS19. The numbers on each branch node indicate the bootstrap support percentage of the corresponding branch.

The phylogenetic trees of glutamate dehydrogenases GdhA and GudB (Figure 2) showed that the GdhA and GudB proteins of *B. altitudinis* AS19 cluster closely with those of *B. pumilus* and *B. safensis*, indicating a close evolutionary relationship among these strains. Specifically, *B. pumilus* GdhA first clusters with *B. safensis* GdhA, suggesting a closer relationship between these two proteins, which is slightly more distant from AS19 GdhA. This pattern is consistent with the phylogenetic relationships inferred from *16S rRNA* sequences. In contrast, the GudB of AS19 first clusters with that of *B. safensis*, and together they form a branch that subsequently groups with *B. pumilus* GudB. This pattern differs from the *16S rRNA*-based phylogeny, suggesting that the evolutionary trajectories of GudB and GdhA may have diverged.

#### 3.1.2. Basic Physicochemical Properties and Structural Analysis of Two Glutamate Dehydrogenases

ProtParam, SignalP 6.0, and TMHMM 2.0 were employed to perform the bioinformatic profiling of *B. altitudinis* AS19 glutamate dehydrogenases. For the protein encoded by *gdhA*, the amino acid length was 456 aa, with a molecular formula of C_2191_H_3440_N_596_O_668_S_22_. Its isoelectric point was 5.09, the instability index was 35.86, the aliphatic index was 83.20, and the average hydropathy value was −0.16. This protein was classified as lipophilic, hydrophobic, and stable. For the protein encoded by *gudB*, the amino acid length was 424 aa, with a molecular formula of C_2075_H_3293_N_561_O_629_S_18_. Its isoelectric point was 5.31, the instability index was 26, the aliphatic index was 89.69, and the average hydropathy value was −0.19. This protein was also classified as lipophilic, hydrophobic, and stable. SignalP and TMHMM analyses revealed that neither GdhA nor GudB contain signal peptides or transmembrane domains, indicating that they are not secreted or membrane proteins (Table 3).

**Figure 3:**
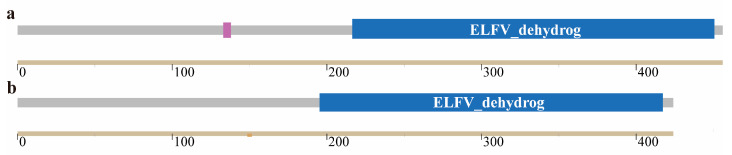
Protein domains of two glutamate dehydrogenases. (**a**) Protein domain of GdhA. Purple indicates low-complexity regions, and blue indicates the conserved glutamate dehydrogenase domain. (**b**) Protein domain of GudB. Blue indicates the conserved glutamate dehydrogenase domain.

The SMART online software was used to predict and analyze the protein domains of the two glutamate dehydrogenases. The results are shown in Figure 3a. GdhA has a functional domain at amino acids 213–454; as shown in Figure 3b, GudB has a functional domain at amino acids 193–422, both of which are typical domains of glutamate dehydrogenase (Figure 3).

**Figure 4:**
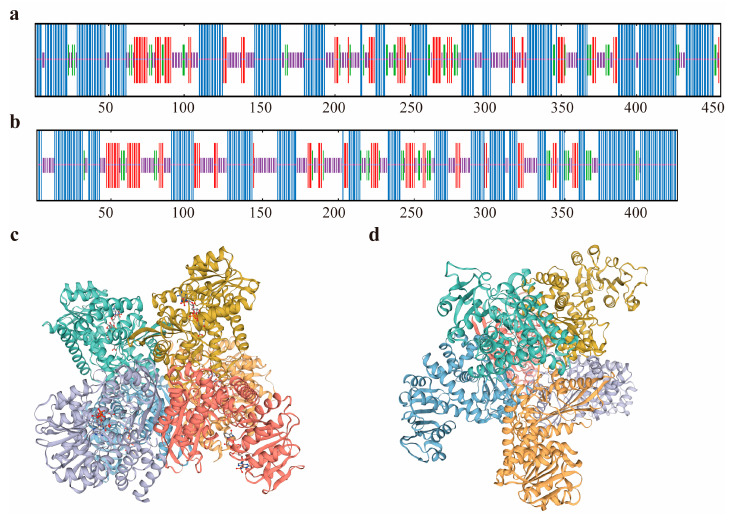
Secondary and tertiary structure predictions of the two glutamate dehydrogenases. (**a**,**b**) Predicted secondary structures of GdhA and GudB proteins. Blue indicates α-helices, purple indicates random coils, red indicates β-sheets, and green indicates β-turns. (**c**,**d**) Predicted tertiary structures of GdhA and GudB proteins. Different colors represent different subunits; the bound cofactor NADPH is shown as sticks.

The secondary and tertiary structure predictions of GdhA and GudB proteins, as determined by SOPMA and SWISS-MODEL software, are depicted in Figure 4. For GdhA protein, the secondary structure is predominantly composed of α-helices (46.93%) and random coils (30.48%), with extended chain structures accounting for 14.04% and β-turns representing the smallest proportion at 8.55% (Figure 4a). Similarly, the GudB protein’s secondary structure is mainly characterized by α-helices (45.75%) and random coils (31.13%), followed by extended chain structures (15.80%) and β-turns (7.31%) (Figure 4b). Homologous tertiary modeling was conducted using 5gud.1.A (glutamate dehydrogenase from *C. glutamicum*) as a template for the GdhA protein (Figure 4c). The sequence similarity between GdhA and the template is 60.85%, within the acceptable range for homologous modeling. GdhA adopts a hexameric structure, primarily consisting of random coils and α-helices, and contains NADPH binding sites within its domain.

For GudB protein, the structure 3k92.1.A (E93K mutant of RocG from *B. subtilis*) was employed as a template (Figure 4d). This template is a NAD-type glutamate dehydrogenase, and its sequence similarity with GudB is 74.23%, fulfilling the criteria for homologous modeling. GudB also forms a hexameric structure, mainly comprising random coils and α-helices. The tertiary structure predictions are in accordance with the secondary structure prediction results.

### 3.2. Results of gdhA and gudB Gene Amplification

**Figure 5:**
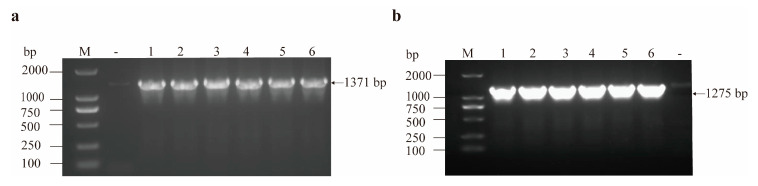
PCR amplification of *gdhA* and *gudB*. (**a**) Electrophoresis map of *gdhA* PCR. M represents DNA marker, “-” represents the negative control, and 1–6 represent the PCR products of *gdhA*. (**b**) Electrophoresis map of *gudB* PCR. M represents DNA marker, “-” represents the negative control, and 1–6 represent the PCR products of *gudB*.

Genomic DNA from AS19 was used as the template for PCR amplification using the designed primers. The PCR products were analyzed using 1% agarose gel electrophoresis. As shown in Figure 5, single bands of the correct size were observed for the target genes: *gdhA* (1371 bp, Figure 5a) and *gudB* (1275 bp, Figure 5b).

### 3.3. Screening and Identification of Recombinant Plasmids

**Figure 6:**
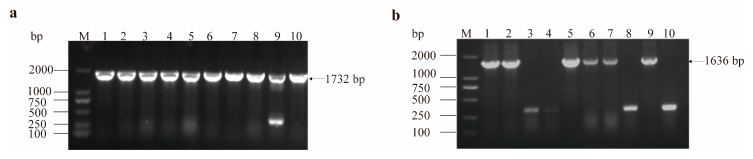
Screening and identification of recombinant plasmids. (**a**) Electrophoresis map of colony PCR for pET28a-*gdhA*. M represents DNA marker, 1–10 are the numbers of the colonies. (**b**) Electrophoresis map of colony PCR for pET28a-*gudB*. M represents DNA marker, 1–10 are the numbers of the colonies.

Following the transformation of the recombinant plasmid, single colonies were selected from LB plates supplemented with 100 µg/mL kanamycin. PCR amplification was conducted using universal primers specific to the pET28a vector. The PCR products were then analyzed by agarose gel electrophoresis (Figure 6a,b). The expected band sizes were observed: the amplified band of pET28a-*gdhA* was approximately 1732 bp (1371 bp of the *gdhA* gene plus 361 bp of the vector sequence) (Figure 6a), and the amplified band of pET28a-*gudB* was approximately 1636 bp (1275 bp of the *gudB* gene plus 361 bp of the vector sequence) (Figure 6b).

### 3.4. Soluble Expression and Purification of Recombinant Proteins

**Figure 7:**
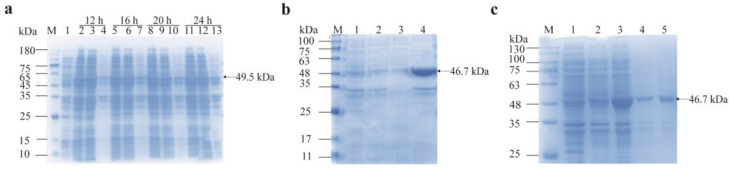
SDS-PAGE analysis of recombinant protein expression. (**a**) Recombinant GdhA expression at 24 °C with 0.2 mmol/L IPTG for various durations. M: Genstar 180 protein marker. Lane 1: Uninduced cells. Lanes 2–4: 12 h induction (cells, soluble fraction, pellet). Lanes 5–7: 16 h induction (cells, soluble fraction, pellet). Lanes 8–10: 20 h induction (cells, soluble fraction, pellet). Lanes 11–13: 24 h induction (cells, soluble fraction, pellet). (**b**): Recombinant GudB expression at 28 °C with 0.2 mmol/L IPTG for 20 h. M: Solarbio Rainbow 180 protein marker. Lane 1: Empty vector pET28a. Lane 2: Uninduced cells. Lanes 3–4: Induced cells (soluble fraction, pellet). (**c**): Recombinant GudB expression at 16 °C with 0.1 mmol/L IPTG for 12 h. M: Solarbio Rainbow 180 protein marker. Lane 1: Empty vector pET28a. Lane 2: Uninduced cells. Lane 3: Induced cells. Lanes 4–5: Induced cells (soluble fraction, pellet).

For GdhA, optimal soluble expression was achieved by inducing cultures at mid-log phase (OD_600_ = 0.6) with 0.2 mmol/L IPTG, followed by a 16 h incubation at 24 °C. Under these conditions, a large amount of protein with a molecular weight of 49.5 kDa was successfully expressed and detected in the soluble fraction (Figure 7a).

For GudB, the initial optimal induction conditions were determined at mid-log phase (OD_600_ = 0.6) using 0.2 mmol/L IPTG, followed by a 20 h incubation at 28 °C. However, the protein was found to form inclusion bodies after ultrasonic disruption (Figure 7b). To achieve soluble expression, the induction conditions were optimized by reducing the temperature and IPTG concentration and shortening the induction time. Ultimately, the target protein with a molecular weight of 46.7 kDa was successfully expressed in the soluble fraction under the conditions of 16 °C, 0.1 mmol/L IPTG, and 12 h (Figure 7c).

**Figure 8:**
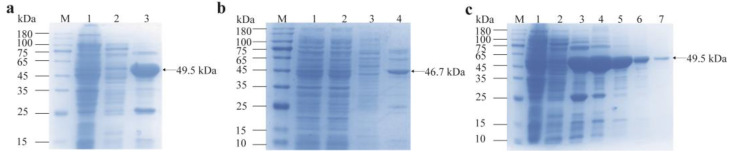
SDS-PAGE analysis of purified recombinant proteins. (**a**) Purified GdhA. M: Genstar 180 protein marker. Lanes 1–3: Flow-through, wash, and eluate fractions, respectively. (**b**) Purified GudB. M: Genstar 180 protein marker. Lanes 1–4: Unpurified sample, flow-through, wash, and eluate fractions, respectively. (**c**) GdhA eluted with gradient imidazole. M: Genstar 180 protein marker. Lane 1: Flow-through. Lane 2: Wash. Lanes 3–7: Eluate fractions with 100 mmol/L, 150 mmol/L, 200 mmol/L, 250 mmol/L, and 300 mmol/L imidazole, respectively.

The recombinant proteins were purified using a Ni-NTA (nickel-nitrilotriacetic acid) affinity column. As shown in Figure 8a,b, the purification process resulted in relatively pure target protein bands. For recombinant GdhA, the optimal purification conditions were identified when the protein was eluted with 300 mmol/L imidazole. Under these conditions, the target protein was enriched to high purity and migrated as a single major band on SDS-PAGE (Figure 8c).

### 3.5. Western Blot Verification and Mass Spectrometry Identification of Recombinant Proteins

**Figure 9:**
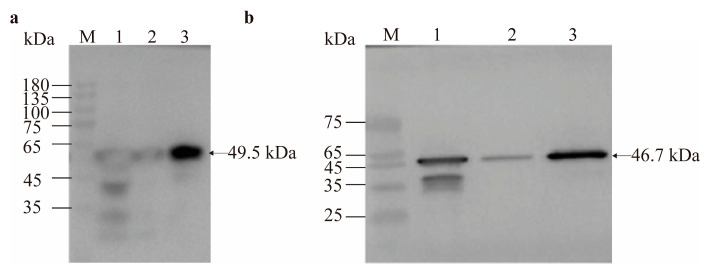
Detection of purified proteins by Western blot. (**a**) Purified GdhA. M represents the Solarbio Rainbow 180 Protein Marker. Lanes 1–3 correspond to the flow-through fraction, washing fraction, and elution fraction, respectively, obtained during the purification process. (**b**) Purified GudB. M represents the Solarbio 180 Protein Marker. Lanes 1–3 correspond to the flow-through fraction, washing fraction, and elution fraction, respectively, obtained during the purification process.

The Western blot analysis demonstrated that the expressed recombinant proteins were specifically bound to mouse anti-His monoclonal antibodies, with distinct specific bands appearing at the expected molecular weights corresponding to the recombinant proteins (Figure 9).

The SDS-PAGE gel strips containing the recombinant protein bands were excised and subjected to mass spectrometry analysis by commercial service providers: Shanghai APTBIO Technology Co., Ltd. (Shanghai, China) and PTM Biolabs Inc (Hangzhou, China). The mass spectrometry results revealed peptide coverage of 72% for GdhA and 82.8% for GudB. Database comparison confirmed that these peptides matched glutamate dehydrogenase encoded by the genes *gdhA* and *gudB*, respectively (Table 4).

**Figure 10:**
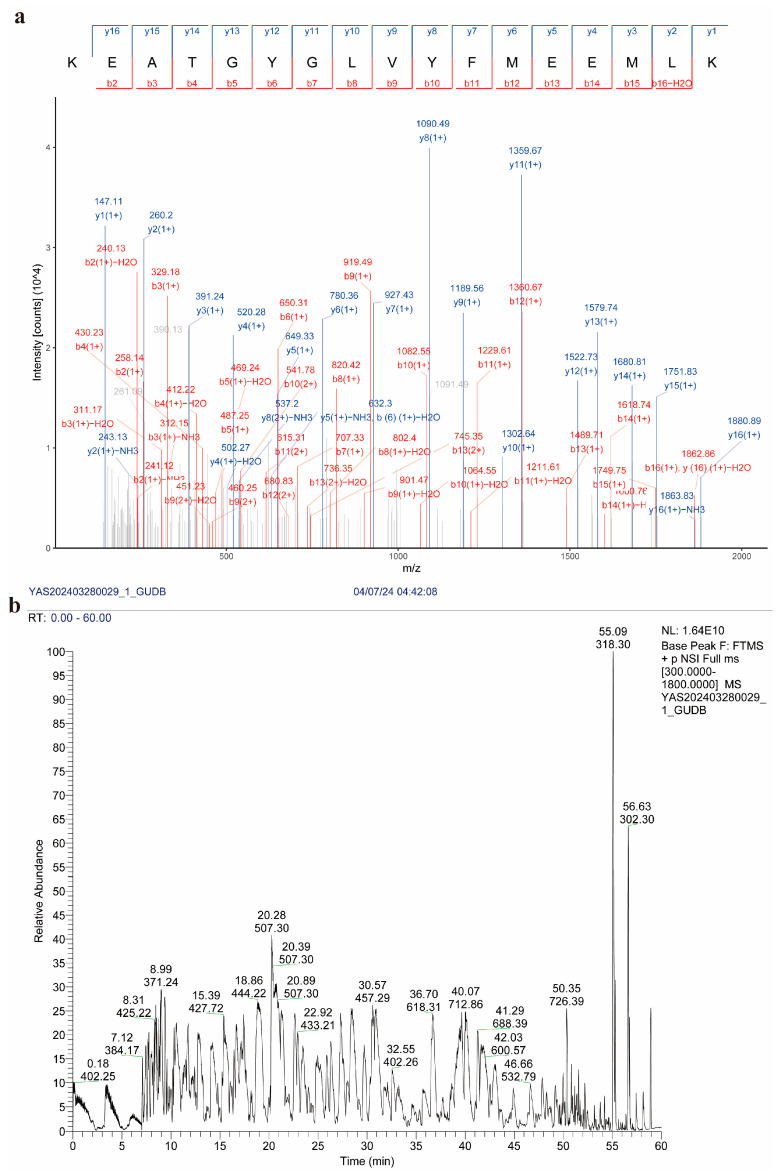
Ion chromatograms of mass spectrometry detection results of recombinant proteins. (**a**) b/y ion chromatogram of mass spectrometry detection of GdhA. (**b**) TIC (total ion current) ion chromatogram during mass spectrometry detection of GudB.

The ion map of the mass spectrometry detection results is presented in Figure 10. Collectively, these findings validated that the recombinant proteins were indeed GdhA and GudB, thereby paving the way for subsequent experiments.

### 3.6. Enzyme Activity Detection of Recombinant Protein

**Figure 11:**
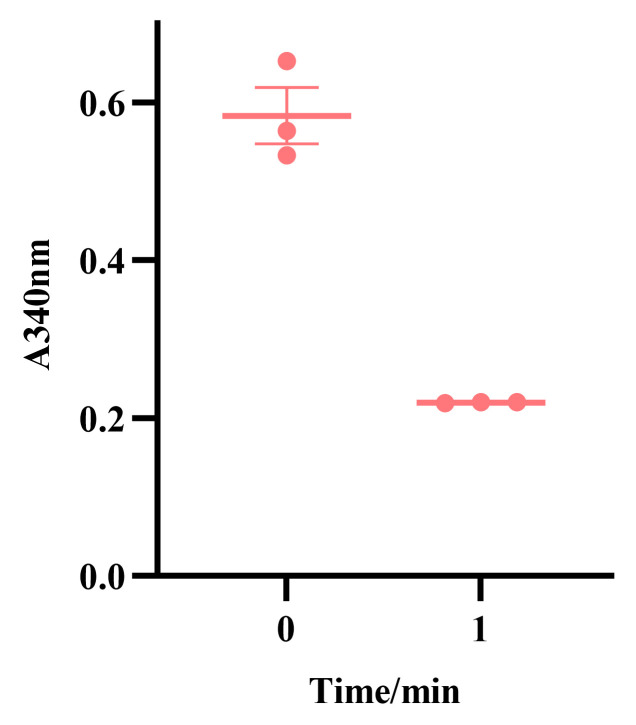
The change in absorbance at 340 nm per minute in the reductive amination reaction system using NADPH as a coenzyme reflects the activity of GdhA.

The recombinant GdhA protein exhibited an enzyme activity of 62.7 U/mg in the reductive amination reaction system when NADPH was used as the coenzyme (Figure 11). In contrast, no enzyme activity was detected for GdhA in other reaction systems tested. For GudB, no enzyme activity was observed in any of the reaction systems examined. These results indicate that the GdhA protein primarily functions in catalyzing the synthesis of glutamate, while GudB did not exhibit detectable catalytic activity under the tested conditions.

## 4. Discussion

Bacteria typically possess only one type of GDH, and it is rare for a single bacterium to harbor two GDHs with distinct characteristics [27]. This study, for the first time, reports the presence of two GDH-encoding genes, *gdhA* and *gudB*, in *Bacillus altitudinis* AS19. These genes are distinct from the GDH-encoding genes *gudB* and *rocG* in *Bacillus subtilis* 168 [26] and the GDH-encoding gene *gdhA* in *Bacillus licheniformis* [25]. Furthermore, the expression of *gdhA* and *gudB* in *B. altitudinis* AS19 was found to be regulated by iron. Currently, studies on GDH in *B. altitudinis* are limited, and the functions of the two GDHs in AS19 require further elucidation.

Obtaining soluble recombinant expression products is crucial for studying the biological functions of proteins. *Escherichia coli* is extensively utilized as a prokaryotic expression host for recombinant proteins [28]. However, due to the absence of auxiliary protein folding machinery, recombinant proteins are susceptible to misfolding, ultimately leading to the formation of inactive inclusion bodies [29]. In prokaryotic expression systems, the soluble expression of proteins is influenced by numerous factors [30]. The selection of host strain, promoter strength, medium composition, culture temperature, and the inherent properties of the protein all significantly impact soluble expression [31]. In the process of protein expression, reduced temperature and inducer concentration can potentially increase the production of soluble proteins [32]. Conversely, high temperatures typically accelerate the synthesis of heterologous proteins, resulting in insufficient time for proper protein folding and the subsequent generation of a large number of inclusion bodies [33,34]. Elevated IPTG concentrations can potentially suppress *E. coli* growth and induce the aggregation of the target protein into inclusion bodies [35].

Previous studies have shown that recombinant expression of glutamate dehydrogenase genes in various strains mostly leads to the formation of inclusion bodies [36]. Chen Lili et al. attempted to reduce the induction temperature, prolong the induction time, and lower the IPTG concentration, but none of these measures increased the solubility of the target protein [37]. Therefore, our study optimized the recombinant expression conditions of two GDHs in *B. altitudinis* AS19. The results showed that reducing the temperature and IPTG concentration and shortening the induction time increased the yield of soluble expression. This approach enabled the soluble expression of the two GDHs, laying the foundation for investigating their physiological functions.

As outlined in the Introduction, GDH catalyzes the pivotal reversible step between α-ketoglutarate and glutamate; the observed functional divergence between GdhA and GudB is therefore entirely reasonable for maintaining overall metabolic balance. GDH can be categorized based on cofactor specificity into three types: NAD(H)-specific, NADP(H)-specific, and dual-cofactor-specific [38]. Typically, NAD(H)-dependent GDH catalyzes glutamate deamination, while NADP(H)-dependent GDH catalyzes glutamate synthesis [24]. Previous studies have shown that the GDH encoded by the *gdhA* gene mainly uses NADP(H) as a coenzyme to assimilate ammonia [24]. In this study, the GdhA of *Bacillus altitudinis* AS19 exhibited high NADPH-dependent activity in synthesizing glutamate. However, the GdhA in other strains also possesses the activity of NADP-catalyzed oxidative deamination [24]. Additionally, the GdhA of *Bacillus natto* has dual coenzyme catalytic activity for both NADPH and NADH [39]. This demonstrates that even though GDH is encoded by the same gene in different strains, its functions and coenzyme preferences can vary significantly.

We also investigated the reason why recombinant GudB was inactive. In this study, the pET28a vector was used to introduce an N-terminal 6×His tag onto both glutamate dehydrogenases. Extensive reports indicate that the His-tag is small and generally does not interfere with the secretion, compartmentalization, folding, or activity of fusion proteins [40,41]. Therefore, we tentatively conclude that the tag will not markedly affect the structure or function of either GDH. To eliminate any potential bias, we will remove the His-tag and conduct systematic control experiments; the results will be reported separately. The *gudB* gene in *B. subtilis* features a direct 9-bp repeat [2], leading to GudB inactivation. In contrast, the *gudB* gene of *B. altitudinis* AS19 lacks this 9-bp repeat sequence. Previous studies have shown that NAD-type GDH is rapidly inactivated at low temperatures [42]. We performed a three-dimensional structure prediction for GudB using SWISS-MODEL, employing the high-resolution crystal structure of NAD-dependent GDH from *B. subtilis* as the template. The resulting model achieved excellent quality metrics that exceed the reliability thresholds established in large-scale benchmarks [23] Therefore, the inactivity of recombinant GudB may be due to protein inactivation after purification and storage under low-temperature conditions following protein expression. Although bioinformatics tools were used to predict the structures and cofactor-binding sites of both GDHs, experimental validation-such as SEC-MALS or X-ray crystallography-will be performed in follow-up studies to confirm their oligomeric states and cofactor binding.

GDH is a member of the amino acid dehydrogenase superfamily and is highly conserved across diverse species. This conservation endows GDH with significant value for evolutionary studies [27]. Phylogenetic analysis revealed that the GdhA and GudB proteins from *B. altitudinis* AS19 are closely related to their counterparts in *B. pumilus* and *B. safensis*, respectively. In the broader context of species evolution, *B. altitudinis*, *B. pumilus*, and *B. safensis* share a close evolutionary relationship. This finding underscores the genetic conservation of kinship at the protein level. It is plausible that the glutamate dehydrogenases from closely related strains may have originated from a common ancestor, thereby sharing similarities in both structure and function.

It has been reported that all glutamate dehydrogenases (GDHs) can be categorized into hexameric GDHs, comprising six identical subunits of about 50 kDa, and tetrameric GDHs, consisting of four identical subunits with a molecular weight near 115 kDa. Bacterial and mammalian GDHs are typically hexameric [3]. In this study, the three-dimensional structures of the two GDHs were determined to be hexameric, with each subunit having a size of around 50 kDa. This finding is in accordance with previous reports.

In this study, bioinformatics approaches were employed to analyze the biological characteristics of two glutamate dehydrogenases (GDHs) from *B. altitudinis* AS19. Following optimization of the expression conditions, soluble expression of these proteins in *E. coli* was successfully achieved. Additionally, the NADPH-dependent enzyme activity of GdhA was detected. These findings lay the foundation for subsequent investigations into the physiological effects, enzymatic properties, and expression regulation of these GDHs.

## Figures and Tables

**Table 1:** Primers used for prokaryotic expression vector construction.

Primer Name	Primers Sequence(5′-3′)	Restriction Sites
*gdhA*-F	CGGGATCCATGTTGACCTTAGATCGAGCAGC	*Bam*H I
*gdhA*-R	CCCAAGCTTTTAAATGACGCCTTGTGCGAT	*Hin*d III
*gudB*-F	CGCGGATCCATTTTAATGGCAGCCG	*Bam*H I
*gudB*-R	CCGGAATTCTTAAATCCAGCCTCTGAATC	*Eco*R I

The underlined sequences are the introduced restriction sites.

**Table 2:** Composition of glutamate dehydrogenase in some *Bacillus* species.

Strains	Glutamate Dehydrogenase Composition	Coding Genes	Similarity with AS19 GdhA (%)	Similarity with AS19 GudB (%)	References
*Bacillus altitudinis* strain AS19	GDH1	*gdhA*	100	30.70	-
	GDH2	*gudB*	30.70	100	
*Bacillus pumilus*	GDH1	*gdhA*	95.83	31.12	-
	GDH2	*gudB*	32.27	99.76	
*Bacillus safensis*	GDH1	*gdhA*	95.18	31.12	[12]
	GDH2	*gudB*	32.04	100	
*Bacillus licheniformis*	GDH1	*gdhA*	70	29.93	[25]
	GDH2	*gudB*	32.31	91.51	
*Bacillus subtilis*	GDH1	*rocG*	30.28	74.76	[26]
	GDH2	*gudB*	31.36	91.80	
*Bacillus amyloliquefaciens*	GDH1	*rocG*	31.47	74.30	-
	GDH2	*gudB*	30.66	91.51	
*Peribacillus tepidiphilus*	GDH1	*gdhA*	73.26	28.95	-
	GDH2	*-*	30.58	76.65	
	GDH3	*-*	31.74	87.76	
*Bacillus mesophilum*	GDH	*-*	31.44	86.82	-
*Bacillus xiapuensis*	GDH1	*gdhA*	71.30	30.07	-
	GDH2	*-*	31.49	75.36	
*Bacillus oleivorans*	GDH	*-*	31.89	86.32	-
*Bacillus smithii*	GDH	*-*	30.16	77.54	-
*Bacillus cereus*	GDH	*gudB*	30.84	84.58	-
*Bacillus thuringiensis*	GDH	*gudB*	31.63	84.58	-
*Bacillus wiedmannii*	GDH	*gudB*	31.40	84.91	-
*Bacillus alkalicellulosilyticus*	GDH	*gdhA*	71.83	29.91	-

Data in the GDH Genes column were derived from NCBI genomic sequence analysis (accession numbers provided in Table A1); the References column exclusively associates with prior studies reporting GDH functionality for each strain.

**Table 3:** Physicochemical properties of two glutamate dehydrogenases.

Physicochemical Properties	GdhA	GudB
Number of Amino Acids	456	424
Molecular Weight (kDa)	49.52	46.74
Isoelectric Point	5.09	5.31
Molecular Formula	C_2191_H_3440_N_596_O_668_S_22_	C_2075_H_3293_N_561_O_629_S_18_
Instability Coefficient	35.86	26
Fat-Solubility Index	83.20	89.69
Average Hydrophilicity Value	−0.16	−0.19
Number of Signal Peptides	0	0
Transmembrane Region	0	0

**Table 4:** Mass spectrometry identification results of recombinant proteins GdhA and GudB.

Target Protein	Protein ID	Protein Name	Gene Name	Sequence Coverage (%)	Number of Peptide	Molecular Weight (kDa)
GdhA	A0A1K2A8A6	Glutamate dehydrogenase	*gdhA*	72.0	40	49.6
GudB	A0A5K1NAB7	Glutamate dehydrogenase	*gudB*	82.8	31	46.7

## Data Availability

The authors confirm that the data supporting the findings of this study are available within this paper.

## Abbreviations

The following abbreviations are used in this manuscript:

GDH	Glutamate dehydrogenase
TCA	Tricarboxylic acid
GS	Glutamine synthetase
GOGAT	Glutamate synthase
TBST	Tris-buffered saline with tween-20
PCR	Polymerase chain reaction
PBS	Phosphate-buffered saline

## Appendix A

**Table A1:** Glutamate dehydrogenase (GDH) genes in representative *Bacillus* strains.

Strains	Glutamate Dehydrogenase Composition	Coding Genes	NCBI Accession
*Bacillus pumilus*	GDH1	*gdhA*	MCY7526718.1
	GDH2	*gudB*	MCY7528696.1
*Bacillus safensis*	GDH1	*gdhA*	AWI38632.1
	GDH2	*gudB*	AWI37181.1
*Bacillus licheniformis*	GDH1	*gdhA*	WP_009329318.1
	GDH2	*gudB*	WP_003183047.1
*Bacillus subtilis*	GDH1	*rocG*	NP_391659.2
	GDH2	*gudB*	NP_390177.2
*Bacillus amyloliquefaciens*	GDH1	*rocG*	WP_013353994.1
	GDH2	*gudB*	WP_013352705.1
*Peribacillus tepidiphilus*	GDH1	*gdhA*	WP_153125470.1
	GDH2	*-*	WP_153126561.1
	GDH3	*-*	WP_153122666.1
*Bacillus mesophilum*	GDH	*-*	WP_151573106.1
*Bacillus xiapuensis*	GDH1	*gdhA*	WP_198508539.1
	GDH2	*-*	WP_100332101.1
*Bacillus oleivorans*	GDH	*-*	WP_097157515.1
*Bacillus smithii*	GDH	*-*	WP_048623896.1
*Bacillus cereus*	GDH	*gudB*	WP_000225173.1
*Bacillus thuringiensis*	GDH	*gudB*	WP_000225172.1
*Bacillus wiedmannii*	GDH	*gudB*	WP_000225171.1
*Bacillus alkalicellulosilyticus*	GDH	*gdhA*	WP_078551346.1
